# Evaluation of Male Circumcision: Retrospective Analysis of One Hundred and Ninety-eight Patients

**DOI:** 10.7759/cureus.4555

**Published:** 2019-04-27

**Authors:** Murat F Ferhatoglu, Abdulcabbar Kartal, Alp Gurkan

**Affiliations:** 1 General Surgery, Okan University Medical Faculty, Istanbul, TUR

**Keywords:** circumcision, complication, child, male

## Abstract

Introduction

Circumcision is the oldest and most frequently used surgical procedure. It dates back to at least 10,000 years from today. The debate on the benefits and necessity of circumcision is ongoing. In this study, we aimed to determine the complications and complication rate of circumcisions occurring in our circumcision clinic and to compare these with the complication rates in the world.

Methods

A total of 198 male patients circumcised between 2011 at 2019 at Bursa State Hospital was enrolled in the presented retrospective study. Demographic data of the patients were assessed and the height and weight of the patients were evaluated according to the child growth standards and weight for age percentile charts for boys of the World Health Organization (WHO). All early or late complications were noted after circumcision.

Results

The mean age of the patients was 93.57±40.12 (2-248) months. The mean follow-up time was 16.32±9.24 (2-35) months. Sixteen patients had bleeding, four patients had a penile hematoma, and 108 patients had penile edema. There is no statistically significant difference in the penile edema occurrence according to the weight of the patients (p=0.58).

Conclusion

Circumcision is a frequently applied procedure. Like any other surgery, perioperative and postoperative complications can be observed. More importantly, a significant number of these complications can be prevented by careful surgery and postoperative care.

## Introduction

Circumcision aims to remove penile foreskin (both penile shaft and inner preputial epithelium) to leave the glans uncovered. It is the oldest and most frequently used surgical procedure [1]. The fact that it is still being made worldwide for religious, traditional, and medical reasons. Although the frequency of this surgical procedure varies according to the region, it is practiced in almost every area of the world. Today, in the United States, circumcision is a commonly performed procedure and half of the men in the United States and Canada. In addition, one-sixth of all men in the world are circumcised. Moreover, circumcision is common in Africa, the Middle East, and Australia while it is relatively rare in East Asia, India, Europe, New Guinea, and South America [2-4].

Although many studies have been conducted on the medical benefits of the circumcision procedure, it is mainly a sociological phenomenon and primarily relies on religious reasons. The origin of circumcision is not known. It is thought that it dates back to at least 10,000 years from today. In the stone-age cave paintings, circumcised male images were found. More prominent findings date back to the 2300s BC. That the Egyptians practiced is evident as the earliest mummies were found to be circumcised [5].

Generally, circumcision complications can be grouped under three main groups as psychological problems, complications related to anesthesia, local anesthesia, and complications related to surgery. Psychological problems, false attitudes in the application of circumcision; families being unable to inform the child sufficiently before the circumcision, insufficiency in psychological preparation, and surgical problems can result in psychological trauma, adverse emotional, and mental effects. Complications related to anesthesia and local anesthesia: apnea, aspiration pneumonia, hypoxia, laryngeal spasm, convulsion, malign hyperthermia, cardiac arrest, methemoglobinemia after prilocaine injection, surgery-related complications: bleeding, infection, necrotizing fasciitis, sepsis, glans, and/or part of the penis amputation, more or less the prepisium excision, trapped penis (secondary phimosis), inclusion cyst, meatit or meatal ulcer, meatal stenosis, mucosal or skin adhesion, urinary retention, penile burns, and iatrogenic hypospadias or urethral injuries, and necrosis of the whole penis [6-7].

In this study, we aimed to evaluate the demographic and medical features and postoperative complications of circumcised patients in a state hospital.

## Materials and methods

A total of 198 male patients circumcised between 2011 and 2017 in Bursa State Hospital were enrolled in the presented retrospective study. Demographic data of the patients were assessed and height, the weight of the patients were evaluated according to the child growth standards and weight for age percentile charts for boys of the world health organization. Six patients operated by general anesthesia and sedoanalgesia were considered to be a small group and excluded from the study. Also, patients having epispadias or hypospadias were not operated and referred to a pediatric surgeon. A physical examination of the patients was performed one day before the surgery. Complete blood count, hepatitis B surface antibody test, hepatitis C antibody test, anti-human immunodeficiency virus-antibody test, and prothrombin time test were preoperatively evaluated. All patients were examined by an anesthesiology doctor one day before the surgery. Informed consent was obtained from families of patients under 18 years of age. Preoperative antibiotic prophylaxis was not applied. The dorsal slit-sleeve technique was applied to all patients. Dorsal penile nerve and the ventral prepicium blockage was administered by local anesthesia (bupivacaine solution two milligrams per kilograms was used after applying 5% lidocaine cream). Bleeding control was achieved by bipolar electrocautery only by coagulation of the veins in the dorsal and ventral vessels if bleeding was observed on the penile frenulum 4/0 polyglactin absorbable sutures used for bleeding control without cauterization. All patients were discharged at the sixth hour of the operation, and they were called to the outpatient clinic at the first and seventh days postoperatively. Postoperative antibiotics were not prescribed; only ibuprofen 7mg/kg per day was prescribed postoperatively. The wound dressings were removed by the surgeon one day after the surgery. The patient was recommended to locally apply a cream containing 0.2% nitrofurazone three times a day around the suture line. All early or late complications were noted after circumcision. Minimal lymphedema and encrustation were not considered a complication.

All procedures are performed according to the Helsinki declaration. SPSS 15.0 for Windows (SPSS Inc., Chicago, IL, USA) was used for the statistical analysis. Descriptive statistics were reported as means ± standard deviation, medians, minimum, and maximum. Categorical variables were reported as numbers and percentages. Differences in ratios were tested for significance by the chi-square test. P-value< 0.05 was considered statistically significant.

## Results

A total of 198 male patients were evaluated. The mean age of the patients was 93.57±40.12 (2-248) months. The mean follow-up time was 16.32±9.24 (2-35) months. The mean height and weight were 120.62±21.55 (57-160) centimeters and, 25.1±10.6 (6-63) kilograms, respectively. The mean body mass index of the patients was 16.44±2.68 (8.54- 28.06) kilograms per square meters. According to the WHO age-weight chart, 253 (21.22%) patients were under the tenth percentile (underweight), 509 (43.5%) patients were between the tenth and ninetieth percentile (normal), and 334 patients (35.1%) were over the ninetieth percentile (overweight) (Table 1).

**Table 1:** Patients' characteristics *According to the World Health Organization's age-weight chart

		Mean± SD (Min-Max.)
Age (months)		93.57±40.12 (2-248)
Follow-up time (months)		16.32±9.24 (2-35)
Weight (kilograms)		25.1±10.6 (6-63)
Height (centimeters)		120.62±21.55 (57-160)
Body mass index (kilograms per square meter)		16.44±2.68 (8.54- 28.06)
Weight ^*^	Underweight	253 (21.22%)
	Average	509 (43.5%)
	Overweight	334 (35.1%)

Of the 242 (20.3%) patients who had phimosis, one (0.08%) patient had an undescended testicle. Also, six (0.5%) had phimosis surgery, 16 (1.34%) had inguinal hernia surgery, and one (0.08%) of the patients had surgery for an undescended testicle before the circumcision operation.

Sixteen patients had postoperative bleeding (six patients were underweight, seven patients were average weight, and three patients were overweight) and no statistically significant difference was found in postoperative bleeding according to weight (p=0.51). Four patients had a penile hematoma (two patients were average weight and two patients were overweight) and 108 patients had penile edema (27 patients were underweight, 45 patients were average weight, and 36 patients were overweight). There is no statistically significant difference in the penile edema occurrence according to the weight of the patients (p=0.58) (Table 2).

**Table 2:** Evaluation of complications based on weight * World Health Organization age-weight chart a Pearson chi-square test; *p<0.05; **p<0.01

		Postoperative bleeding n (%)	Penile edema n (%)
Weight^*^	Underweight	6 (37.5)	27 (25)
	Average	7 (43.75)	45 (41.6)
	Overweight	3 (18.75)	36 (33.3)
	p	^a ^0.51	^a ^0.58

Twelve of 16 patients having bleeding were managed by a tight bandage; the other four patients needed surgical exploration for bleeding. None of the patients had a severe infection or penile burn due to cautery injury.

## Discussion

The debate on the benefits and necessity of circumcision is ongoing. In the literature, reports show that circumcision causes deterioration of some parameters of the sexual function and some parameters cause a positive change. Also, some publications show that it does not create a significant difference [2,6,8]. In men with circumcision, the incidence of sexually transmitted diseases, including acquired immune deficiency syndrome, is lower [9-11]. Moreover, the development of penile cancer is less common in circumcised men. The development of cervical cancer in the partners of circumcised men is also less [12-13]. Besides, circumcision prevents, as expected, phimosis, paraphimosis, and problems such as balanitis [14-15]. It is evident that such findings can be obtained through long-term observations. The limitation of our study was that the follow-up time was up to 35 months.

There are publications in the literature that are positive and opposite to circumcision. Complication rates depend on multiple factors, including anatomic abnormalities, medical comorbidities, surgical technique, and patient age. This paper will deal with the most common complications and methods to manage them. In Western literature, it was common for those who said circumcision should be routinely practiced before now this rate has changed against circumcision [16]. There are complications from bleeding due to a gender change and even death was reported [17]. Gee and Ansell's report of 5882 case series had a significant complication rate of 0.2% [16]. Significant complications of the circumcision procedure are life-threatening bleeding, systemic infection, complete excision of the penis skin, and amputation of the whole penis. The minor complication rate was also 7.4% [18]. The risk of mortality was also two in a million [16]. An accurate and complete preoperative evaluation, focusing on bleeding history and birth history, is imperative. Proper selection of patients based on age and anatomic considerations, as well as appropriate sterile surgical technique, are critical to prevent future circumcision-related adverse events. Our complication rate was 10.7%, but none of them were significantly life-threatening complications or lifelong disabilities.

Penile edema was the most observed complication, with a rate of 9.06%. With the increasing age of the patient, the occurrence of complications becomes more frequent. Bleeding becomes more common during the “mini-puberty" of infancy that begins at four weeks of age and extends to three months of age. This is thought to be due to a hormonally mediated increase in penile and prepuce size and vascularity [4]. In a recent prospective observation-based study of 583 neonatal circumcisions, Banieghbal reported only two minor bleeding complications requiring sutures. Both occurred in infants aged three weeks. Based on the use of the Neonatal Infant Pain Scale, he further reported that the ideal timeframe for a "pain-free" circumcision is during the first week of life [19]. This is further supported by Horowitz and Gershbein who reported zero complications in 98 infants circumcised with a Gomco clamp in their first month [20].

While complications such as bleeding and infection are seen in all methods, the incidence of some complications is affected by the method used. For example, glandular injury is more common when using the Mogen clamp technique or penile shaft injuries are reported more frequently when the Gomco clamp is used [18]. The glans amputation seen using the Mogen clamp suggests that there may be a common injury mechanism. This mechanism may be an inadequate liberalization of balanopreputial adhesions around the rim [21]. Therefore, it is essential to completely remove the adhesions before excision to reduce the risk of glans amputation in the absence of the glans. It is thought that the glans amputation seen when using the Mogen clamp technique is due to inadequate liberalization of the balanopreputial adhesions around the frenulum. To reduce the risk of penile glans injuries in the Mogen technique, it is essential to remove adhesions before skin excision completely. We used the dorsal slit-sleeve technique in all of the patients, and 0% of a significant complication rate was observed. This method requires crushing and division of the inner and outer preputial layers dorsally. The slit is extended to the corona. This enables the prepuce to be freed entirely and excised, under direct vision. This technique was effectively used in our center with a good outcome. 

## Conclusions

Although circumcision is a frequently applied procedure, like any other surgery, perioperative and postoperative complications can be observed. More importantly, a significant number of these complications can be prevented by careful surgery and careful postoperative care. Before the circumcision, an excellent preoperative physical examination, history, and laboratory tests are required. If these conditions are considered, we think that the probability of developing complications is very low.
